# The application, value, and impact of outcomes research in animal health and veterinary medicine

**DOI:** 10.3389/fvets.2022.972057

**Published:** 2022-11-29

**Authors:** Diana M. A. Dewsbury, David G. Renter, Barry J. Bradford, Keith D. DeDonder, Marnie Mellencamp, Natalia Cernicchiaro

**Affiliations:** ^1^Center for Outcomes Research and Epidemiology and Department of Diagnostic Medicine and Pathobiology, College of Veterinary Medicine, Kansas State University, Manhattan, KS, United States; ^2^Department of Animal Science, College of Agriculture and Natural Resources, Michigan State University, East Lansing, MI, United States; ^3^Latham BioPharm Group, Elkridge, MD, United States; ^4^Zoetis, Outcomes Research, Parsippany, NJ, United States

**Keywords:** outcomes research, outcomes, animal health, veterinary medicine, veterinary research

## Abstract

Outcomes research is a relatively recent field of study in animal health and veterinary medicine despite being well-established in human medicine. As the field of animal health is broad-ranging in terms of animal species, objectives, research methodologies, design, analysis, values, and outcomes, there is inherent versatility in the application and impact of the discipline of outcomes research to a variety of stakeholders. The major themes of outcomes relevant to the animal health industry have been distilled down to include, but are not limited to, health, production, economics, and marketing. An outcomes research approach considers an element of value along with an outcome of interest, setting it apart from traditional research approaches. Elements of value are determined by the stakeholders' use of products and/or services that meet or exceed functional, emotional, life-changing, and/or societal needs. Stakeholder perception of value depends on many factors such as the purpose of the animal (e.g., companion vs. food production) and the stakeholder's role (e.g., veterinarian, client, pet-owner, producer, consumer, government official, industry representative, policy holder). Key areas of application of outcomes research principles include comparative medicine, veterinary product development, and post-licensure evaluation of veterinary pharmaceuticals and/or biologics. Topics currently trending in human healthcare outcomes research, such as drug pricing, precision medicine, or the use of real-world evidence, offer novel and interesting perspectives for addressing themes common to the animal health sector. An approach that evaluates the benefits of practices and interventions to veterinary patients and society while maximizing outcomes is paramount to combating many current and future scientific challenges where feeding the world, caring for our aging companion animals, and implementing novel technologies in companion animal medicine and in production animal agriculture are at the forefront of our industry goals.

## Introduction

Outcomes research has been defined as research concerned with outcomes, or end results, and entails the application of clinical and population-based methods to optimize healthcare practices and interventions (1). Albeit considered a formal discipline in human medicine for several decades (2), it remains a relatively young area in veterinary medicine (3) and the animal health industry. Outcomes and value are key foundations of this field, with outcomes research being at the intersection of outcome optimization and quantification of the elements of value. Of recent relevance to animal health are outcomes such as quality of life (4), individual preference (5), and cost-effectiveness (6). Commonly, value is associated with a reduction in cost; however, cost and value are not always synonymous (7). Value can be represented by many factors that address functional, emotional, life-changing, and/or societal needs (7). The perceived value of those outcomes ultimately depends on the stakeholder, whether a pet-owner, farm manager, government official, or industry representative.

Currently, the veterinary and animal health sectors are facing substantial challenges including an increase in the pet population, increase in the costs of veterinary care and products, increased financial limitations of owners and managers, and pressure to increase food supply (8, 9). These challenges have exacerbated the ongoing reduction in access to large and companion animal veterinary care, especially in communities that already have inadequate access to veterinary services (10). We theorize that outcomes research principles and methods that consider client preferences, costs, and health risks can aid decision-making by veterinary practitioners and animal health professionals to minimize the use of clinical practices or interventions that are medically ineffective or not cost-effective while maximizing resources. In this perspective article, we briefly discuss the principles and methods of outcomes research, illustrate current examples of this field in the animal health sector, and describe the application of human healthcare outcome research trends and novel technologies in the animal health industry.

### Brief introduction of principles and methods of outcomes research

The key themes of outcomes vital to the animal health industry can be distilled to health, production, economics, and marketing, although there are often points of overlap among them. Health and production outcomes are many and well-known in the companion and large animal veterinary fields. Varying based on the targeted species, economic outcomes in companion animals typically reflect cost to the pet-owner, willingness to pay, and overall affordability of veterinary care on an individual animal basis. As food animals are considered a commodity, livestock producers are most interested in an overall low cost and/or an increased net-return on investment. Marketing outcomes, instead, include any characteristic that can be used to market a product and/or service that demonstrates a clear value proposition (11), such as a more affordable alternative or a non-monetary metric. Measuring the safety and efficacy of administration of oral and topically administered fluralaner in canines with sarcoptic mange (12), demonstrating canine acceptance of two bioequivalent carprofen chewable tablets (13), or evaluating equine treat palatability and associated owner preferences (14), are examples of studies with marketing outcomes. Intuitively, economic outcomes commonly overlap with health, production, and marketing outcomes. For instance, the use of gamithromycin for metaphylaxis in stocker steers was associated with better performance measured by average daily gain (production outcome), lower morbidity (health outcome), and greater net-return per head (economic outcome) compared to the competitor product—ceftiofur crystalline free acid (marketing outcome) (15). Evaluating key outcomes and value relevant to the decision makers, while optimizing efficiency, demonstrating comparative efficacy, and maximizing resources demonstrates the quintessence of outcomes research.

The overarching goal of healthcare providers, either human or veterinary, should be to achieve projected outcomes and improve value for patients (16). Value, however, is not the same as cost. Instead, value refers to focusing on quality of service, as opposed to volume, and on maximizing outcomes that matter to patients relative to the incurred costs (16). Metrics of value are not mutually exclusive and multiple metrics may be represented within a study while addressing more than one stakeholder need. In fact, Almquist et al. (7), state that the more elements of value that are conveyed, “the greater a customer's loyalty and the higher the company's sustained revenue growth”. The four types of needs—functional, emotional, life-changing, and societal (7)—used to represent value are determined by the stakeholder. Potential functional needs may include interventions and/or services that reduce time and/or effort (e.g., ease of administration), avoid hassle (e.g., acceptability, palatability), reduce risk of disease, or integrate easily into daily routines (7). Value can also address psychosocial or emotional needs such as improving quality of life, contributing to overall increase in wellness, providing therapeutic value, and being readily available to the stakeholder when needed. Lastly, life-changing and social impact needs that contribute to perceived value reflect aspects of a product/service that may provide hope or an organization that considers charity and gives back, respectively (7). Philanthropy efforts can be seen in the work done by The Zoetis Foundation which has committed to providing $35 million dollars over 5 years to support communities and their animals, veterinary training, veterinary student scholarships, and to care for animals impacted by disasters (17). Additional philanthropic efforts have been put forward by Elanco's Healthy Purpose™ initiative to advance the well-being of animals, people, and the planet (18), and the MerckHelps™ assistance program to provide Merck medicines and vaccines for free to people who qualify (19). A decision to purchase a product from these companies may be driven, in addition to the potential use of the product, by the customer's desire to make a difference.

For a review on the origins and evolution, as well as principles and application of outcomes research in veterinary medicine, the reader may refer to Cernicchiaro et al. (3).

### Application of human healthcare outcome research trends and novel technologies in the animal health industry

Every year, the International Society for Pharmacoeconomics and Outcomes Research (ISPOR), the international organization for the advancement of policy, science and practice of the discipline of outcomes research, publishes the “Top 10 Health Economics and Outcomes Research (HEOR) Trends” (20). Though focused on human healthcare, these trends are also shaping the themes and the methodologies applied to the animal health industry, especially in the areas of drug and healthcare pricing, digital technologies and advanced analytics, which were identified among the 2022–23 top trends (20). Discussions pertaining to the cost and price of products and services have dominated the veterinary field for years. The price of drugs and transparency of costs incurred for veterinary treatment is essential for building trust with pet-owners (21) and providing a spectrum of care (22). As per the National Health Council (23) “value assessment advises whether a health service (e.g., drug, device, and surgery) should be used, and if so, how it is best used in the healthcare system, and which patients are most likely to benefit from it.” Most value assessment frameworks evaluate the health benefits and risks of using a treatment or technology, but they can also assess costs and other wider impacts on a population (23). Several value assessment approaches, such as cost-effectiveness analysis, are used to inform product pricing. Cost-effectiveness models evaluate health effects and costs associated with treatment (24), hence offering a better option than a cost-benefit analysis, which simply monetizes a health effect while ignoring important aspects associated with treatment such as quality of life (24, 25). Similarly, decision analytical models, which provide evidence to guide decision making by utilizing mathematical techniques to synthesize data comparing expected costs and consequences of potential decisions (26), can be used to evaluate long-term outcomes as well as economic impacts (27). Survival extrapolation that includes general population mortality has been recently utilized (28) for evaluating oncology treatments in companion animals. While some of the trends observed in human and veterinary healthcare overlap, a disconnect in methodologies remains, and thus, a tremendous benefit could be gained by translating and applying these methods to current issues in animal health.

The use of digital technologies in animal health is also of recent interest for areas like willingness to pay for veterinary telemedicine (29) and assessing traceability of live animals and their products (30). The trend of precision medicine, or personalized medicine, is a growing field in human (20) but also in veterinary medicine. Precision medicine utilizes big data (20) with predictive technologies and advanced analytics to identify the best treatment on an individual basis. Recently, the use of technology demonstrated successful bovine respiratory disease treatment of individual cattle upon arrival that was similar in overall effect to traditional metaphylaxis (31). This predictive technology reduced antibiotic use in the cattle production environment after determining whether an individual animal required treatment or not (31) rather than a subjective decision to treat an entire population at the time of arrival; hence, technology has the potential to improve antimicrobial stewardship and subsequently reduce the costs associated with treatment (31).

Real-World Evidence (RWE) in decision making has prevailed as a top trend in recent years (20). The application of RWE by veterinary pharmaceutical companies to supplement the product development licensure process could be transformative in reducing the time to bring veterinary products to market. As defined by the Food and Drug Administration (FDA), Real-World Data (RWD) are data relating to patient health status and/or the delivery of healthcare, whereas RWE is the analysis of RWD regarding usage, benefits, and/or risk of a medical product (32). The advantages and limitations of the use of RWD and RWE in human healthcare product development have been reviewed in detail, and overall, show great promise to accelerate product development as the results and findings are more indicative of how the product performs in the real-world (33). As recently reported in human vaccine licensure (34), the use of RWD and RWE, could also expedite the regulatory approval process of vaccines and therapeutics in animal health, such as when using client-owned animals to prove concept of new animal drugs after the completion of necessary safety studies. A recently published guidance by the Food and Drug Administration, Center for Veterinary Medicine (FDA-CVM) describes how RWD and RWE can be used to support regulatory decisions relative to the effectiveness of new animal drugs (35). Despite having a greater external validity, there are concerns on relying solely on RWD and RWE for regulatory approval, as they lack the internal validity of randomized controlled trials. Likewise, not all objectives are amenable to RWE, including the development of pharmaceutical products against Biosafety level 3 and 4 pathogens, the production of novel compounds that are not yet made in mass production facilities, or measuring outcomes such as methane release or feed intake in dairy cattle. The abundance, diversity and size of RWE, which includes post-marketing studies, patient registries or physician reports, among other data sources, presents challenges to conventional analytic methods (20). In addition to knowledge translation and synthesis methods (e.g., systematic reviews, meta-analysis) which can assist the appraisal and synthesis of high volumes of data, machine learning techniques and artificial intelligence offer ways to analyze these data and provide clinical prediction and treatment, among other applications (36, 37). Early adoption of robust RWD collection and synthesis methods, coupled with the use of technology and automation would enable the timely generation, and at a reasonable cost, of stakeholder- and clinically-relevant outcomes in the veterinary pharmaceutical research and development, as well as in other relevant animal health areas.

### Current examples and future directions of outcomes research in the animal health industry

The utility of outcomes research in the areas of comparative medicine, veterinary product development, and evaluation is demonstrated in the following sections.

#### Comparative medicine

In comparative medicine, human and veterinary medicine are considered as “two branches of one medicine”, as they share similar problems and approaches to solutions for humans and animals (38). In human pharmaceutical and biological development, laboratory animal models are utilized to prove a concept, and to demonstrate efficacy, or safety, before utilizing non-human primate and/or human research subjects in clinical trials (39). Traditionally, murine models are used, however, inherent weaknesses and limitations have been demonstrated over the years with findings in rodents not translating well into human medicine—“mice are not men” (40). When choosing which animal model is most appropriate, there are scientific, regulatory, and animal welfare considerations to contemplate prior to designing research trials (40). In recent years, alternative laboratory animal models—including canine, feline, and swine models—have demonstrated extreme value in expediting advancements in human healthcare research and product development. Specifically, the use of purpose-bred canine and feline animal models has led to successful approval of many therapies for many rare, yet extremely debilitating and lethal, genetic diseases in humans (40, 41). These animals also offer more efficient and externally valid models than rodents for certain tumors, such as lymphoma and leukemia. These ailments are not only more common in canines than humans, they progress in the same aggressive manner (42), leading to the utilization of canines as pre-clinical models to evaluate new, and modify current, therapies for human hematopoietic neoplasia (42).

Animal models are also commonly used in the development of human and animal vaccines for infectious disease prevention and management (43). Swine have demonstrated their usefulness as a biomedical model for humans in terms of metabolic, cardiovascular, digestive, and bone diseases (44–47). Lelovas and colleagues (44), discussed lessons learned over the last two decades of cardiovascular research utilizing animal models and the comparative anatomy of swine to humans, deeming minipigs to be the most appropriate animals for cardiovascular research. Commercial swine have also been used in biomedical research, most commonly in nutrition and physiology studies, where growing swine proved a suitable model for human metabolic studies in food research (45).

The longevity of companion animals has been substantially extended thanks to the improvements made in veterinary care as well as the advances in dietary and medical technologies. Attempts to understand the phenomenon of aging, as a way to increase the healthy lifespan of people and animals, has led to innovative initiatives such as the Dog Aging Project (48), which aims to understand how genetic, lifestyle, and environmental determinants influence aging, and how that information can be used to inform medical breakthroughs for humans and dogs. McCune and Promislow (49) suggested that dogs, given their proximity to humans in terms of genetic variation, pathophysiological processes, shared environment, and also because of their relatively short lifespan, can serve as sentinels for observing the beneficial and detrimental effects of environmental factors. Maximizing healthy aging and quality of life outcomes, as well as advancing efficacious, safe and affordable treatments and services for aging pets should become future priorities of the veterinary and animal health sectors.

Outcomes research in comparative medicine utilizes animal models for veterinary and human medicine evaluation, refinement, and ultimately approval of human and veterinary therapies and interventions, and considers relevant outcomes addressing primarily *functional* and *emotional* values, leading the way toward an overall improvement of the quality and longevity of human and animal life.

#### Veterinary product development, licensure, and post-licensure evaluation

In the veterinary field, pharmaceutical products and biologicals rely solely on research to provide pivotal information under regulatory guidance in order to receive approval for licensure from the respective agencies (50). Traditionally, the product development and licensure processes require many different experimental trials to demonstrate proof of concept, efficacy, and safety. It is estimated that the time required to develop a veterinary pharmaceutical product is 5–15 years, from discovery to licensure, with costs potentially exceeding $100 million (50). Conversely, the time to develop and license a veterinary vaccine is ~5–8 years with costs estimated between $50 and $100 million (50). Time to licensure is generally shorter for veterinary products compared to human products, and the input costs and profits are also reduced. The COVID-19 pandemic highlighted the ability to rapidly develop a safe and effective human vaccine using mRNA technology (20). Although this example was an unusual pathway to market which emerged under extreme conditions, this rapid time to market amidst a global emergency shows there is room to improve efficiency in the licensure process for veterinary and human medicine fields moving forward. Post-licensure evaluation is commonly conducted to compare against other licensed products to yield marketing information and/or to supplement technical bulletins; however, additional time, labor, expertise, animals, and costs are required to generate supplemental data to prove value and the competitive edge over rival products. A major difference between human and animal health development/approval is that because of the importance of being approved/listed in insurance/formularies (i.e., market access), when human health products go through the approval process, they include a comprehensive assessment of cost/value, which is traditionally not implemented in animal health (unless a company wishes to do so post-approval). Through the use of outcomes research, the product development and licensure pathways have the potential to more efficiently utilize resources by addressing efficacy or effectiveness outcomes, as well as added value such as ease of administration, storage or temperature requirements, affordability, and/or net-return on investment.

## Discussion

Outcomes research has been a formal discipline for over two decades, although more formally implemented in the animal health industry in recent years. The foundation of outcomes research is comprised of two elements, outcomes and value. This added component of value is what sets the discipline of outcomes research apart. Value can be measured by many characteristics that satisfy four primary needs: emotional, functional, societal, and life-changing, with the perceived value being determined by the stakeholder. Although decision-makers/stakeholders and values can differ in animal health, human health outcomes research continues to pave the way through shaping outcomes research based on current trends, and through the development and implementation of novel methodologies. Though easily transferable to the animal health industry, work is still needed in interpreting novel technologies utilized in human healthcare, such as long-term cost-effectiveness and decision analytical models. Additionally, the use of Real-World Data and Real-World Evidence to aid and/or supplement in the product licensure process in animal health could transform veterinary product development. While underrepresented in the literature during development due to confidentiality and intellectual property issues, outcomes research plays a role in licensure of veterinary products during the development phase as well as post-licensure as the focus of marketing veterinary products is through comparative efficacy and value proposition. Future efforts of outcomes research in the animal health industry should focus on bridging the gap between novel methodologies and technologies utilized in human health and translating their efforts into the context of animal health.

## Data availability statement

The original contributions presented in the study are included in the article/supplementary material, further inquiries can be directed to the corresponding author.

## Author contributions

DD and NC conceived and outlined and wrote the manuscript. DR, BB, KD, and MM contributed to the article, reviewed, and approved the final version of the manuscript.

## Funding

This study was funded by the Center for Outcomes Research and Epidemiology, the Department of Diagnostic Medicine and Pathobiology, and the College of Veterinary Medicine at Kansas State University.

## Conflict of interest

Author KD was employed by Latham BioPharm Group. Author MM was employed by Zoetis. The remaining authors declare that the research was conducted in the absence of any commercial or financial relationships that could be construed as a potential conflict of interest.

## Publisher's note

All claims expressed in this article are solely those of the authors and do not necessarily represent those of their affiliated organizations, or those of the publisher, the editors and the reviewers. Any product that may be evaluated in this article, or claim that may be made by its manufacturer, is not guaranteed or endorsed by the publisher.
